# Engineering of lysin by fusion of antimicrobial peptide (cecropin A) enhances its antibacterial properties against multidrug-resistant *Acinetobacter baumannii*

**DOI:** 10.3389/fmicb.2022.988522

**Published:** 2022-09-26

**Authors:** Md Maidul Islam, Dooyoung Kim, Kyeongmin Kim, Su-Jin Park, Samia Akter, Jeongah Kim, Seunghyeok Bang, Shukho Kim, Jungmin Kim, Je Chul Lee, Chang-Won Hong, Minsang Shin

**Affiliations:** ^1^Department of Microbiology, School of Medicine, Kyungpook National University, Daegu, South Korea; ^2^Functional Bio-material Research Center, Korea Research Institute of Bioscience and Biotechnology (KRIBB), Jeongeup, South Korea; ^3^Department of Physiology, School of Medicine, Kyungpook National University, Daegu, South Korea

**Keywords:** *Acinetobacter baumannii*, cecropin A, endolysin, antibacterial activity, synergistic effect

## Abstract

Most clinical isolates of *Acinetobacter baumannii*, a nosocomial pathogen, are multidrug-resistant (MDR), fueling the search for alternative therapies. Bacteriophage-derived endolysins have potent antibacterial activities and are considered as alternatives to antibiotics against *A. baumannii* infection. Gram-negative bacteria possess outer lipid membrane that prevents direct contact between the endolysins and the cell wall. We hypothesized that the fusion of antimicrobial peptide (AMP) with endolysin could help to reduce bacterial endolysin resistance and increase antimicrobial activity by membrane permeability action. Accordingly, we fused cecropin A, a commonly used AMP, with the N-terminus of AbEndolysin, which enhances the bactericidal activity of the chimeric endolysin. The bactericidal activity of cecropin A-fused AbEndolysin increased by at least 2–8 fold for various MDR *A. baumannii* clinical isolates. The *in vitro* bactericidal activity results also showed higher bacterial lysis by the chimeric endolysin than that by the parental lysin. The engineered AbEndolysin (eAbEndolysin) showed synergistic effects with the beta-lactam antibiotics cefotaxime, ceftazidime, and aztreonam, and an additive effect with meropenem and imipenem. eAbEndolysin had no cytotoxic effect on A549 cell line and rescued mice (40% survival rate) from systemic *A. baumannii* infection. Together, these findings suggest the potential of lysin therapy and may prompt its use as an alternative to antibiotics.

## Introduction

Pathogens with resistance to three or more antimicrobial agents are called multidrug-resistant (MDR) bacteria that have recently become a major public health concern (Li et al., 2015; Thadtapong et al., 2021). *Acinetobacter* species is hospital-oriented bacterial pathogens responsible for several epidemics worldwide (Almasaudi, 2018). Patients with vulnerable health conditions in the intensive care units are highly susceptible to infection by carbapenem-resistant *Acinetobacter baumannii* (Hu et al., 2022), which is listed as a top priority critical pathogen by the World Health Organization and requires new antimicrobial agents (Peleg et al., 2008; WHO, 2017). *A. baumannii* causes various healthcare-associated infections and may acquire and transfer antibiotic-resistant determinants, making it an MDR (Vrancianu et al., 2020). New and effective measures are urgently needed to control MDR bacterial infections, such as *A. baumannii*.

Antibiotic resistance has prompted the search for alternative treatment methods. Bacteriophage-derived endolysins could serve as an alternative to antibiotic treatment (Oliveira et al., 2018). Endolysins are released in the late stage of the bacteriophage infection cycle and have peptidoglycan-hydrolyzing enzyme-containing domain called catalytic domains and sometimes contain second domain for substrate recognition and binding (Oliveira et al., 2012). Endolysins have several advantages compared to phages, including modular structure, wide host spectrum, low bacterial resistance and synergy with other antibiotics (Ghose and Euler, 2020; Gondil et al., 2020). Owing to the absence of an outer membrane in gram-positive bacteria, endolysins can directly lyse the cell wall (Chang, 2020). However, gram-negative bacteria contain an outer lipid membrane that prevents direct contact between endolysin and peptidoglycan for degradation. OM permeabilizers, such as ethylene-diamine-tetraacetic acid (EDTA) or transmembrane peptides, are generally used to enhance endolysin activity (Briers and Lavigne, 2015). Recombinant and engineered endolysins have been successful against both gram-positive and -negative bacterial pathogens, such as methicillin-resistant *Staphylococcus aureus* (MRSA), *Listeria*, *A. baumannii*, and *Pseudomonas aeruginosa* (Chen et al., 2021; Murray et al., 2021; Abdelkader et al., 2022). Modification of endolysins with antimicrobial peptides (AMPs) bypasses the necessity of adding EDTA or organic acids for increasing the effectiveness of endolysins in gram-negative bacteria (Yang et al., 2015). AMPs are naturally occurring peptides with precise mechanisms of action and immunomodulatory features that make them a substitute for conventional antibiotics (Mookherjee et al., 2020). Cecropins are amphipathic peptides secreted by insects that facilitate outer membrane permeabilization owing to their hydrophobic and cationic characteristics (Hancock and Chapple, 1999). Cecropin A was first reported by Boman et al. in 1981, and its antibacterial activity was shown to be specific only for bacteria (Steiner et al., 1981). Previously, we described the endolysin protein AbEndolysin of phage Ab1656-2, comprising N-terminal N-acetylmuramidase and C-terminal peptidoglycan-binding domains, and has antibacterial activity against clinical *A. baumannii* strains (Kim et al., 2021). Phage Ab1656-2 was isolated from the MDR biofilm-forming *A. baumannii* 1656–2 strain recovered from a Korean hospital (Lee et al., 2008). Its antibacterial activity was determined using the microdilution method, and the overall minimum inhibitory concentrations (MICs) for different clinical isolates was 125–250 μg/ml (in the presence of co-factor Zn^2+^; Kim et al., 2021).

We hypothesized that AMP fusion with AbEndolysin could improve the lytic potential of the parental endolysin against MDR bacteria. In this study, we selected the AMP cecropin A to create a chimeric protein with AbEndolysin. By constructing a chimeric endolysin using cecropin A and AbEndolysin at the N-terminus, we explored the lytic potential of the engineered AbEndolysin (eAbEndolysin) against MDR *A. baumannii* strains. Several previous studies have suggested combining endolysin with antibiotics to take advantage of their synergistic action to kill MDR *A. baumannii* (Thummeepak et al., 2016; Abdelkader et al., 2022). A previous study showed that the synergistic effect was observed only with colistin; however, in our study, we tested antibiotics belonging to the β-lactam family (cefotaxime, ceftazidime, aztreonam, meropenem, and imipenem), which affect bacterial cell wall biosynthesis. Lastly, we investigated the cytotoxic effect and *in vivo* protection of eAbEndolysin in a mouse infection model.

## Materials and methods

### Bacterial strains, plasmids, and bacteriophage

The bacterial strains and plasmids used in the present study are listed in Supplementary Table S1. *Acinetobacter baumannii* ATCC 17978 was obtained from a laboratory collection, and clinical isolates were obtained from the Kyungpook National University Hospital National Culture Collection for Pathogens (KNUH-NCCP). Luria–Bertani (LB) broth and Mueller–Hinton broth (MHB) with or without 1.5% agar were used to culture the bacterial strains at 37°C or 18°C with ampicillin and trimethoprim when needed. *Escherichia coli* DH5α and BL21 (DE3) Star strains were used for cloning and protein expression, respectively.

### Antibiotic susceptibility testing

The susceptibility of clinical *A. baumannii* isolates to antimicrobial agents was determined using the broth dilution method, as described earlier (Jung et al., 2020), and analyzed according to the Clinical and Laboratory Standards Institute (CLSI) guidelines (CLSI, 2022). Susceptibility to ampicillin, aztreonam, cefotaxime, ceftazidime, cefepime, imipenem, meropenem, piperacillin, tazobactam, ticarcillin/clavulanic acid, and colistin was measured. *E. coli* ATCC 25922 and *Pseudomonas aeruginosa* ATCC 27853 were used as the quality control strains. Antimicrobial susceptibility tests were performed in triplicates.

### AbEndolysin-fused cecropin A cloning, overexpression, and purification

Cecropin A is a 37-residue AMP and was cloned at the N-terminus of AbEndolysin. Cecropin A and AbEndolysin nucleotide sequences were supplied in supplementary section which was PCR-amplified separately and combined using overlap extension PCR with the forward primer 5′-CGGGGGCGGTGGTGGCGGC ATGGCTAGCAAATGGAAACTGTTTAAAAAA-3′ and reverse primer 5′-GTTCTTCTCCTTTGCGCCCTATATAACAACTCGATTGGCGATC-3′ designed for ligation-independent cloning (Aslanidis and de Jong, 1990). The full-length amplicon was digested with Sma1 (Thermo Fisher Scientific, Waltham, MA, United States) restriction enzyme and the vector pB4 derived from the pET21a plasmid (Novagen, Darmstadt, Germany; Kim et al., 2017) was also digested with same enzyme. After restriction enzyme digestion, both PCR product and pB4 vector were linked by treatment with T4 DNA polymerase (New England Biolabs, Beverly, MA, United States) to construct the recombinant plasmid pB4-cecropin A-AbEndolysin and spreading ampicillin containing agar plate. The new construct had the feature of T7 promoter, lac operator, RBS, maltose-binding protein (MBP)-tag, tobacco etch virus (TEV) protease recognition and cleave site in frame with cecropin A fused AbEndolysin and 6xHis-affinity tag, respectively. The correct clones were verified using PCR and Sanger sequencing (Macrogen Inc., Seoul, Korea). Subsequently, the chemo-competent *E. coli* BL21 (DE3) Star strain was transformed with the sequence-verified pB4-cecropinA-AbEndolysin for overexpression and purification.

Recombinant endolysin was overexpressed as previously described (Kim et al., 2021). Briefly, a single colony from the transformed agar plate was cultured overnight in the LB broth supplemented with 100 μg/ml ampicillin at 37°C. Fresh culture was prepared by 1/100 dilution in 1 l LB broth and cultured at 37°C until an optical density (OD_600_) at 600 nm ranging from 0.5 to 0.6 was obtained. At this culture condition, isopropyl-β-d-thiogalactopyranoside (IPTG) was added at a final concentration of 0.150 mM to induce protein synthesis and the culture flask was incubated at 18°C overnight under shaking (150 rpm) conditions. Next, the cells were pelleted using centrifugation (Beckman JA-14 rotor, Brea, CA, United States) and suspended in 40 ml buffer (20 mM Tris–HCl pH 7.5, 0.5 M NaCl). A sonicator was used to break down the cell wall; the soluble fraction was collected using centrifugation (16,000× *g* for 30 min at 4°C), and the cell debris was removed by filtering through a 0.45-μm filter (Millipore Corporation, Bedford, MA, USA). The filtered sample was then applied to the Ni-NTA resin in the HisTrap™ HP 5-mL column (GE Healthcare, Little Chalfont, United Kingdom) and chromatographically purified using an FPLC chromatography system (Pharmacia, Stockholm, Sweden). TEV protease was used to cleave MBP from His-tagged eAbEndolysin in the dialysis buffer (20 mM Tris, pH 7.5, 500 mM NaCl, 10 mM β-mercaptoethanol, and 1 mM EDTA) at 4°C overnight. After dialysis, the samples were applied to a second Ni-NTA column, and then the fractions were collected using the FPLC system and concentrated using the Amicon Ultra-4 Centrifugal Filters Ultracel-10 K (Millipore Corporation). Dialysis of purified protein was performed in 20 mM Tris–HCl, pH 7.5 and 100 mM NaCl buffer and this was used as final buffer throughout the experiments. The purity of the eluted protein was measured using sodium dodecyl sulfate polyacrylamide gel electrophoresis (SDS–PAGE) in which gel was stained with Coomassie brilliant blue; their concentrations were measured using a NanoDrop 2000 Spectrophotometer (Thermo Fisher Scientific) and stored at −80°C until use.

### Minimum inhibitory concentration and minimum bactericidal concentration measurement

The antibacterial activity of AbEndolysin or eAbEndolysin was determined using the broth microdilution method in round-bottomed 96-well microplates. Twelve *A. baumannii* clinical isolates within six sequence types ST191 (KBN10P04948, KBN10P06070), ST208 (KBN10P04320, KBN10P04969), ST229 (KBN10P04598, KBN10P06126), ST369 (KBN10P04596, KBN10P04621), ST451 (KBN10P05231, KBN10P05986), and ST784 (KBN10P04697, KBN10P05713) were used to test the bactericidal activity. Bacterial strains were grown exponentially and diluted with MHB to obtain a concentration of 4 × 10^6^ colony-forming unit (CFU/mL), and 100 μl bacterial suspension was added to each well. Then 100 μl of purified AbEndolysin or eAbEndolysin (250–3.9 μg/ml final concentration) with buffer was added and two-fold serially diluted to each well. Finally, 200 μl bacteria and endolysin mixture was incubated at 37°C for 18 h. Bacterial mixture without endolysin was used as the control. The MIC values were determined as the lowest concentration that completely inhibited bacterial growth by visual inspection. Minimum bactericidal concentration (MBC) was determined after confirming the MIC results using the same clinical isolates and 96-well plates. After mixing well, a replica plater for 96-well plate (Sigma-Aldrich Korea, Seoul, Korea) was used to collect the samples from the test plate and spot on an LB agar plate and incubate at 37°C for 18 h. The lowest endolysin concentration that inhibited growth on agar plates after incubation for 18 h was defined as the MBC. All assays were performed in duplicates.

### Field-emission scanning electron microscopy

Circular glass slides were placed on 6-well cell culture plates after flame sterilization, coated with poly-L-lysine, and further sterilized under ultraviolet light for 1 h. Next, 2 ml LB broth was added to each well and inoculated with a 1:100 diluted overnight bacterial culture and fresh culture up to OD_600_ = 0.5 in a shaking incubator at 37°C. The glass slides were transferred to a new 6-well plate containing 2 ml fresh LB broth. Subsequently, 125 μg/ml AbEndolysin or eAbEndolysin was added to each well and incubated in shaking condition for 1 h at 37°C. Bacteria without treatment with AbEndolysin or eAbEndolysin was used as negative control. Next, without endolysin or AbEndolysin- or eAbEndolysin-treated slides were washed with phosphate-buffered saline (PBS) and fixed by dehydration using 20, 50, and 70% ethanol for 15 min each and 100% ethanol for 40 min. The samples were coated with Pt/Au for 90 s and imaged by adjusting the resolution and magnification using a field-emission scanning electron microscope (Hitachi SU8220).

### Effects of temperature, pH, salinity and serum on endolysin activity

To study the effect of temperature on endolysin activity, 100 μl (50 μg) AbEndolysin or eAbEndolysin was pre-treated at 4–80°C and added to 100 μl bacterial suspension (4 × 10^6^ CFU/ml). Thermostability experiment was conducted in 20 mM Tris–HCl pH 7.5 and 100 mM NaCl buffer. After incubation for 30 min, the mixture was serially diluted and plated on an LB agar plate. The same volume of the bacterial mixture and buffer without endolysin was used as the negative control. The number of viable bacteria (CFUs) on agar plates was evaluated after incubation at 37°C for 18 h. Antibacterial activity was expressed as a decrease in viable bacterial counts compared to the control. To evaluate the effect of different pH on endolysin activity, 50 μg AbEndolysin or eAbEndolysin was resuspended in 20 mM Tris–HCl at pH 5.5–9.5. After incubation for 30 min with the bacterial suspension (4 × 10^6^ CFU/ml), the mixture was serially diluted, and antibacterial activity was measured as described above. The same volume of the bacterial mixture and buffer without endolysin was used as the negative control. The tolerance of endolysin to different NaCl concentrations was measured after resuspending AbEndolysin or eAbEndolysin in buffers containing 0–700 mM/l NaCl in 20 mM Tris–HCl (pH 7.5). Next, 100 μl endolysin (50 μg) and 100 μl bacterial suspension (4 × 10^6^ CFU/ml) were incubated at 37°C for 30 min and plated. The antibacterial activity was measured as described above. The influence of serum on the lytic activity of AbEndolysin or eAbEndolysin was measured at various human serum concentrations ranging from 1 to 20% in buffer with different concentration of AbEndolysin or eAbEndolysin (62.5–250 μg/ml). After mixing endolysin, serum and equal numbers of *A. baumannii* ATCC 17978, the mixture was incubated at 37°C for 2 h and serially diluted for plating on an LB agar plate including trimethoprim antibiotic. The lytic activity was measured by the reduction in the viable cells (CFU/mL) enumerated after incubation at 37°C for 18 h with the lysin compared to negative control. Samples without AbEndolysin or eAbEndolysin was used as negative control. Serum dependent lytic activity of eAbEndolysin was also determined using 10% serum and 250 μg/ml AbEndolysin or eAbEndolysin using 6 different clinical isolates from six ST-types following the same protocol described above. All experiments were performed in duplicates. Data are expressed as the mean ± standard deviation.

### Synergy testing

The conventional checkboard broth dilution method was used to determine the synergy between eAbEndolysin and different antibiotics (cefotaxime, ceftazidime, aztreonam, meropenem, and imipenem). Briefly, 0–31.2 μg/ml eAbEndolysin and different antibiotic concentrations were two-fold serially diluted in 96-well plates horizontally and vertically, respectively. *A. baumannii* 17978 cells were added to each well (4 × 10^6^ CFU/ml). The MICs were determined by visual inspection of cells that did not grow after incubation at 37°C for 18 h and bacterial growth (OD_600_) was measured using a microplate reader (Molecular Devices, Sunnyvale, United States). The fractional inhibitory concentration (FIC) for eAbEndolysin or antibiotics was calculated by dividing the MIC of the two in combination with the MIC of each antibiotic alone. The FIC index (FICI), the sum of the FICs of each eAbEndolysin and antibiotic, was used to confirm the interaction between the two drugs. Checkboard assay for six different clinical *A. baumannii* (KBN10P06070, KBN10P04969, KBN10P06126, KBN10P04621, KBN10P05231 and KBN10P05713) isolates using same 5 antibiotics tested before was also performed as described above. The FICI was considered as follows: synergistic effect ≤0.5, additive >0.5 to >1 (Pillai et al., 2005).

### *In vitro* cytotoxicity assay

To determine the cytotoxicity of eAbEndolysin, the human alveolar cell line A549 was used. First, the cells were suspended in 100 μl (5 × 10^3^ cells/well) and seeded in a 96-well plate. After incubation at 37°C and 5% CO_2_ for 24 h, different eAbEndolysin concentration (final concentration: 250–62.5 μg/ml) in 10 μl was added to the cells and incubated for 24 h. PBS and 1% Triton X-100 were used as the positive and negative controls, respectively. Then, 10 μl of the Cell Counting Kit-8 (Dojindo Molecular Technologies Inc., United States) solution was added to each well. After incubation at 37°C in a 5% CO_2_ incubator for 2 h, the OD_450_ was measured using a microplate reader (Molecular Devices). The experiment was carried out twice, and data are presented as the mean ± standard deviation.

### *In vivo* mouse protection assay using *Acinetobacter baumannii* systemic infection

All experimental animal infection procedures were approved by the Animal Care Committee of Kyungpook National University, South Korea. Briefly, 6-week-old female BALB/c mice were housed at five mice per case. To promote infection, 150 mg/kg cyclophosphamide was intraperitoneally (IP) administered prior to bacterial infection (−4 and − 1 d) to induce neutropenia (van Faassen et al., 2007). The mice were IP injected (200 μl) with *A. baumannii* ATCC 17978 (2 × 10^8^ CFU) to induce systemic infection when required. The mice were divided into four groups (five mice per group) for IP injection as follows: PBS control group, infection with *A. baumannii* ATCC 17978 group, 125 μg/ml eAbEndolysin safety test group, and 125 μg/ml eAbEndolysin + infection with *A. baumannii* group. After 0.5 h infection, the mice were treated with eAbEndolysin by IP injection and monitored for survival once daily for 5 days. Another mice survival experiment was also conducted using eight groups (five mice per group) for IP injection as follows: PBS control group, infection with *A. baumannii* ATCC 17978 group, 125 μg/ml eAbEndolysin safety test group, 125 μg/ml, and 250 μg/ml eAbEndolysin + infection with *A. baumannii*, 125 μg/ml AbEndolysin safety test group, 125 μg/ml and 250 μg/ml AbEndolysin + infection with *A. baumannii.* The number of live and dead animals was inputted into GraphPad Prism (GraphPad Software, San Diego, California, United States), and a survival curve was generated. Similar to survival assay, a neutropenic mice systemic infection model was used to observe the bacterial loads in different organs. After IP injection of mice with *A. baumannii* ATCC 17978 (2 × 10^8^ CFU) and similar treatment group with eAbEndolysin as used before, mice were euthanized 12 h after injection. The number of bacteria in the blood, lungs, liver, and spleen samples was enumerated on agar plates, by serial dilution method and CFU counting.

## Results

### Antibiotic resistance profile of clinical *Acinetobacter baumannii* isolates

The antibiotic resistance patterns of 12 *A. baumannii* clinical isolates were determined against antibiotics commonly used to gram-negative bacterial infection (ampicillin, aztreonam, cefotaxime, ceftazidime, cefepime, imipenem, meropenem, piperacillin, tazobactam, ticarcillin/clavulanic acid, and colistin) The MIC results showed that all the tested clinical isolates were resistant to the 10 tested antibiotics and only susceptible to colistin (Supplementary Table S2).

### Engineering of AbEndolysin

**Figure 1:**
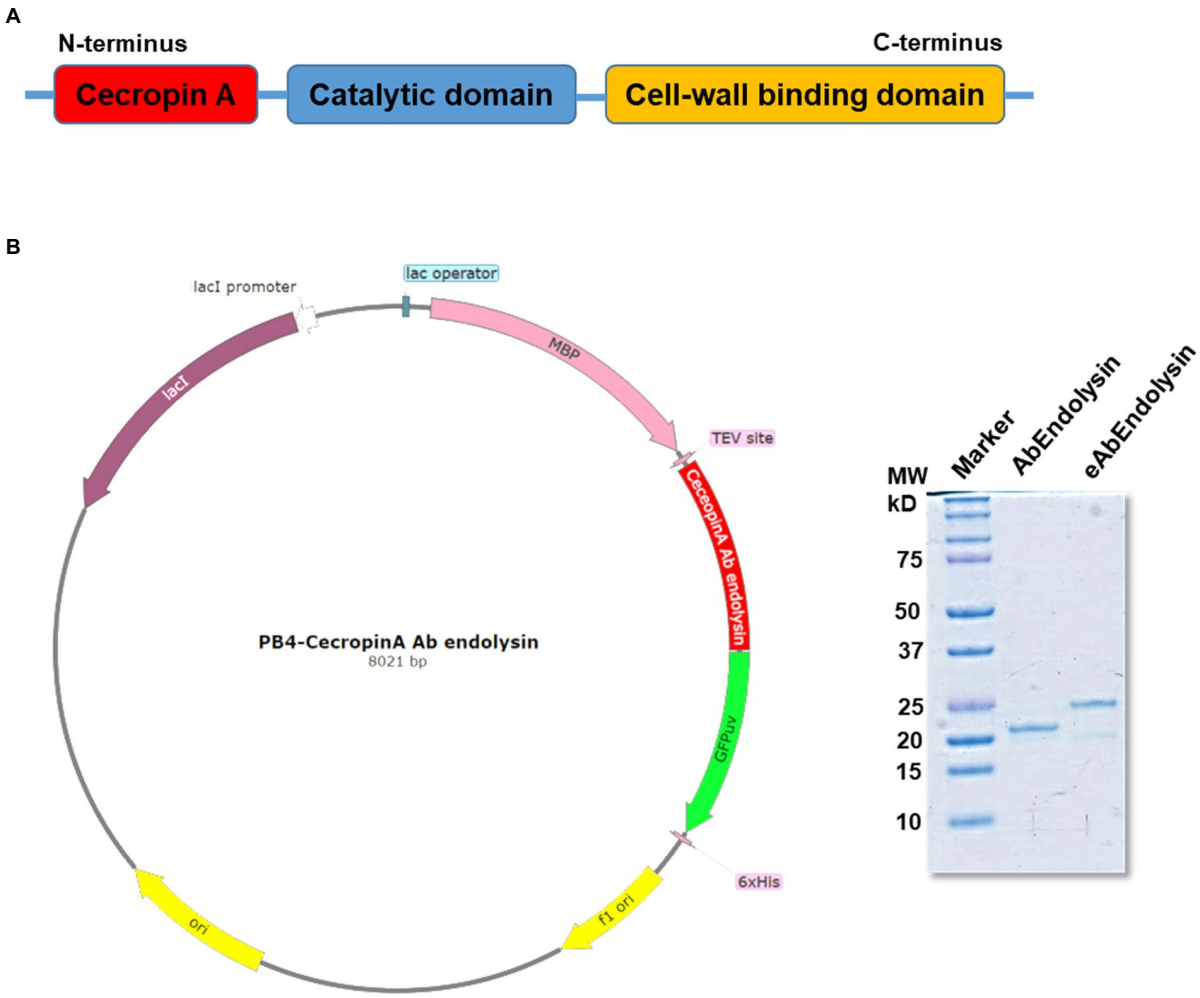
Cloning and purification of cecropin A-fused AbEndolysin. **(A)** Fusion of cecropin A at N-terminus of AbEndolysin comprising two domains: N-terminal catalytic domain (sky blue color) and C-terminal cell-wall binding domain (yellow color). **(B)** Overall scheme used for the purification of eAbEndolysin. Purified recombinant endolysin was separated using a 12% sodium dodecyl sulfate polyacrylamide gel. Lane 1: marker, Lane 2: 22 kDa AbEndolysin, and Lane 3: 26.5 kDa eAbEndolysin.

Previously, we have reported that bacteriophage Ab1656-2 (GenBank accession number MZ67574) consists of AbEndolysin, a putative endolysin with the N-terminal N-acetylmuramidase and C-terminal peptidoglycan-binding domains (Kim et al., 2021) Purified AbEndolysin showed different degrees of antibacterial activity against several clinical *A. baumannii* isolates in the presence of Zn^2+^ (Kim et al., 2021). A previous study showed that lysin engineering by fusion with the AMP cecropin A enhances antimicrobial activity against *A. baumannii* (Gerstmans et al., 2020; Chen et al., 2021). To increase the activity of AbEndolysin, a 37-amino acid sequence of cecropin A, an AMP was fused at the N-terminus of AbEndolysin (Figures 1A,B). The full-length sequence of AbEndolysin with N-terminal fusion of cecropin A and C-terminal His-tag was successfully cloned into the IPTG-inducible expression plasmid pB4. The recombinant plasmid was expressed in *E. coli BL21* (DE3) after IPTG induction at 18°C and purified. Both purified proteins had the respective molecular mass of AbEndolysin (22 kDa) and eAbEndolysin (26.5 kDa) confirmed using SDS–PAGE (Figure 1B).

### eAbEndolysin has higher antibacterial activity than parental lysin

**Figure 2:**
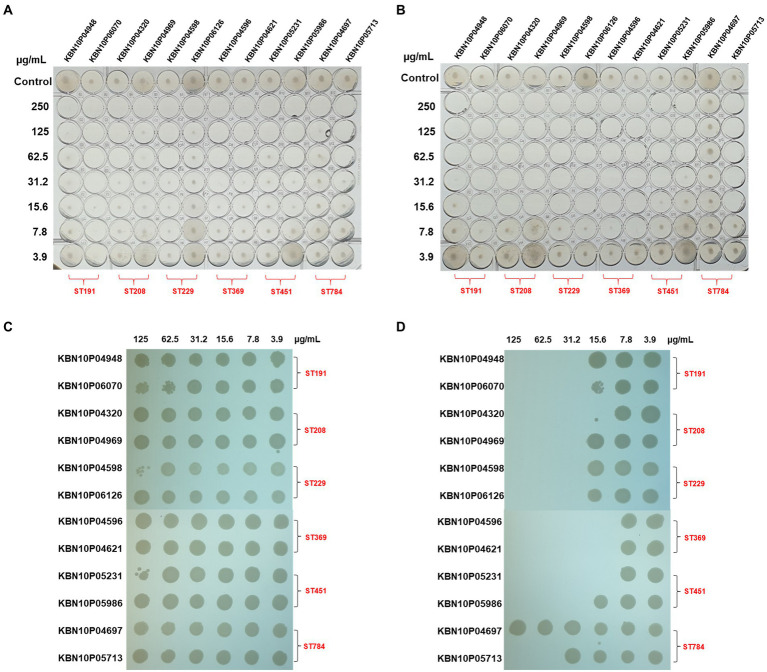
Antibacterial activity of AbEndolysin and eAbEndolysin. **(A,B)** Antimicrobial activity of AbEndolysin **(A)** and eAbEndolysin **(B)** against multidrug-resistant (MDR) *Acinetobacter baumannii* clinical isolates (ST191, ST208, ST229, ST369, ST451, and ST784) were measured by minimum inhibitory concentration (MIC). AbEndolysin was serially diluted two-fold from the highest concentration (250 μg/ml) to confirm the inhibitory concentration. Fresh bacterial culture in Mueller–Hinton broth (MHB) was used as control. **(C,D)** Minimum bactericidal concentration (MBC) of AbEndolysin **(C)** and eAbEndolysin **(D)** against the same set of bacteria.

We hypothesized that cecropin A fusion may increase membrane permeability, thereby enhancing the antibacterial potency of AbEndolysin. To confirm this hypothesis, we tested the antibacterial activities of AbEndolysin and eAbEndolysin against 12 MDR *A. baumannii* clinical isolates with six different sequence types using the microdilution method. The MIC of eAbEndolysin increased 8-fold against KBN10P04969, KBN10P06126 isolates, 4-fold against KBN10P04948, KBN10P04320, KBN10P04598, KBN10P04621, KBN10P05231, KBN10P05713 isolates, 2-fold against KBN10P06070, KBN10P04596 isolates and unchanged against KBN10P05986, KBN10P04697 isolates, compared to parental AbEndolysin, respectively. Overall, the MIC of eAbEndolysin ranged from 15.6 μg/ml to 31.2 μg/ml for most tested isolates except for one isolate, ST784, whereas that of the parental AbEndolysin ranged from 62.5 μg/ml to 125 μg/ml for most tested isolates (Figures 2A,B). In addition, the MBC results also supported the MIC results and confirmed the enhanced antibacterial activity of the engineered lysin than that of the parent lysin (Figures 2C,D).

### *In vitro* bactericidal effect of eAbEndolysin

**Figure 3:**
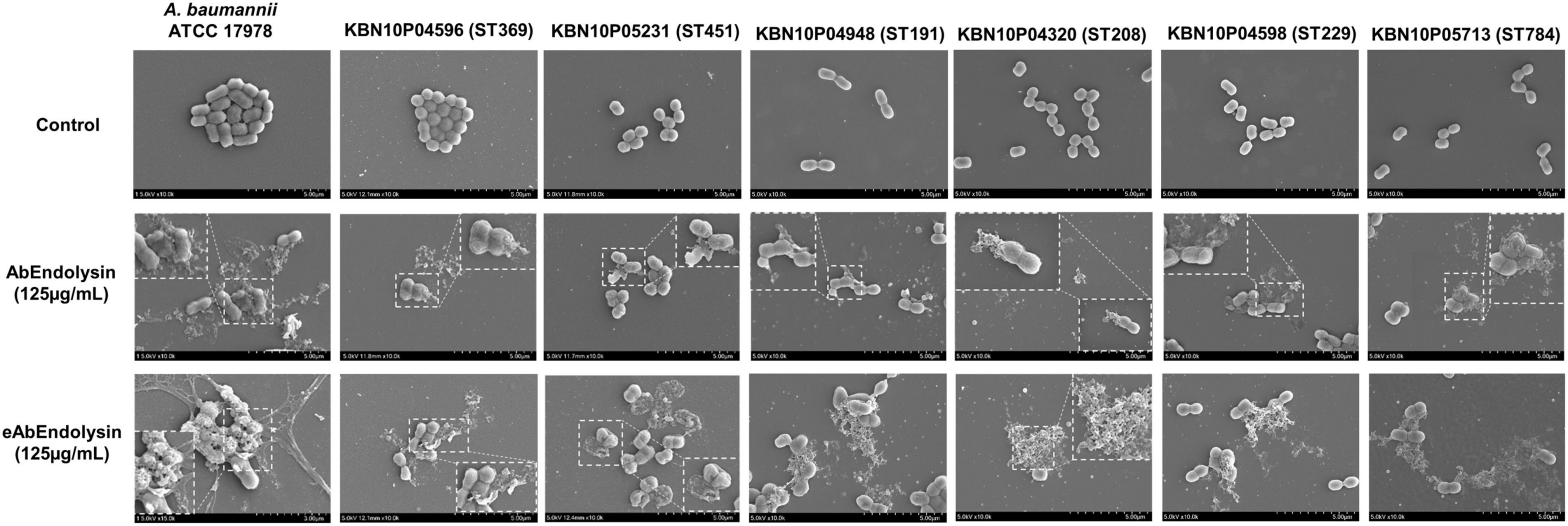
*In vitro* bactericidal effect of eAbEndolysin. Representative scanning electron microscopy (SEM) images of multidrug-resistant *A. baumannii* ATCC 17978 and clinical isolates (ST191, ST208, ST229, ST369, ST451, and ST784) after incubation with AbEndolysin, eAbEndolysin (125 μg/ml), and PBS were visualized using a field-emission scanning electron microscope. The scale bar corresponds to 5 μm.

The *in vitro* bactericidal effects of AbEndolysin and eAbEndolysin were determined using field-emission scanning electron microscopy (FE-SEM). After incubating *A. baumannii* 17978 and six clinical isolates of six different STs with the same amount of AbEndolysin and eAbEndolysin for a certain period, bacterial cell membranes were lysed by both endolysins (Figure 3). Untreated cells showed smooth and bright surfaces with no apparent cell debris, whereas endolysin-exposed bacterial cells showed damaged cells with cell debris and collapsed cell structure. FE-SEM images showed that the same concentration of eAbEndolysin showed a higher cell lysis capability than that of parental lysin for all tested isolates (Figure 3).

### Influence of temperature, pH, salinity and human serum on bactericidal activity of eAbEndolysin

**Figure 4:**
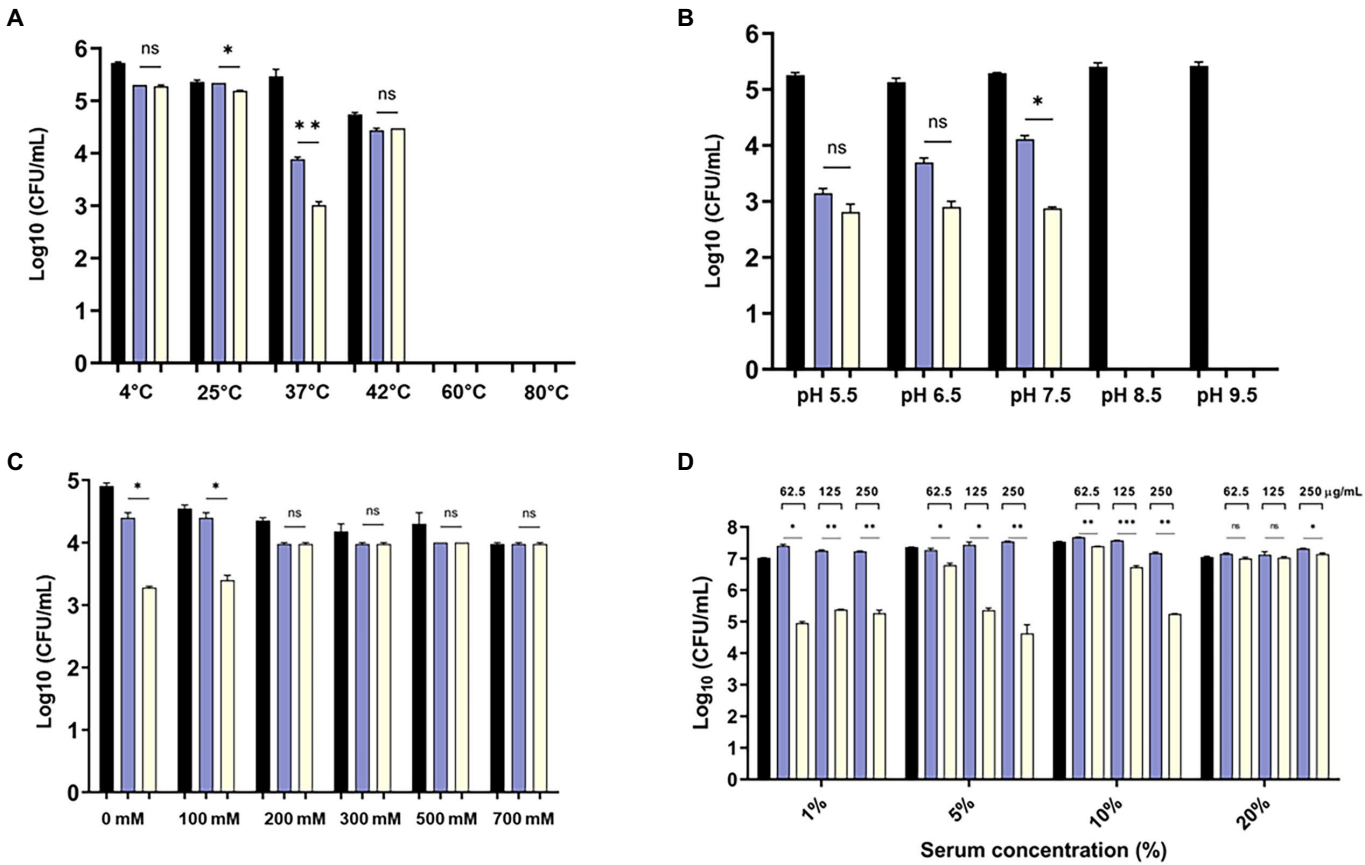
Effect of temperature, pH, salinity and human serum on the lytic activity of eAbEndolysin. **(A)** Effect of temperature on AbEndolysin and eAbEndolysin stability after incubation at 4–80°C. **(B)** Effect of pH values on AbEndolysin and eAbEndolysin activity after incubation at pH 5.5–9.5. **(C)** Effect of salinity on AbEndolysin and eAbEndolysin activity in 0–700 mM/l NaCl solutions. **(D)** Activity of AbEndolysin and eAbEndolysin in presence of human serum (1–20%). The number of log10 CFU/mL was determined through plating 10-fold serial dilution in a bactericidal assay. Data are expressed as the mean ± SD. Experiments were performed independently in duplicate. Statistical significance was determined by t-test. (*p < 0.05; **p < 0.01; ***p < 0.001 and ns- not significant). Black indicate untreated endolysin, sky blue indicate treatment with AbEndolysin and yellow indicate treatment with eAbEndolysin.

The bactericidal activity of the modified endolysin was tested under different conditions, such as temperature, pH, and salinity. The bactericidal activity at 4–80°C was tested, and the results showed that modified lysin had high bactericidal activity at 4–42°C, but no activity was detected above 60°C (Figure 4A). There was significant increase in bactericidal activity of eAbEndolysin compared to native lysin at 37°C. The antibacterial activity of endolysin was tested at pH 5.5–9.5, and the results demonstrated high lytic activity at pH 5.5–7.5. High pH reduced the endolysin activity (Figure 4B). Thus, the optimum pH for the bactericidal activity of eEndolysin was 7.5 at which there was significant increase of bactericidal activity compared to native lysin. Our results of tolerance to salinity demonstrated that the engineered endolysin showed higher antimicrobial activity at low NaCl concentrations (100–0 mM) than at high NaCl concentrations compared to control and parental lysin (Figure 4C). Enzymatic activity was reduced at high NaCl concentrations (200–700 mM) and there was no difference between engineered and native lysin activity. The activity of AbEndolysin or eAbEndolysin was tested in different concentrations (1, to 20%) of human serum. The eAbEndolysin showed good bactericidal activity in 1, 5 and 10% serum compared to AbEndolysin. Although there was about 2 log-fold increase in bactericidal activity of eAbEndolysin in 1% serum, it does not depend on eAbEndolysin concentration (Figure 4D). The killing efficacy increases in 5 and 10% serum based on increased concentration of eAbEndolysin from 62.5–250 μg/ml, respectively. No significant activity was observed at 20% serum concentration. In case of clinical isolates, serum dependent higher bactericidal activity of eAbEndolysin was observed in ST229, ST451 and ST784 isolates compared to native lysin (Supplementary Figure 2). We also analyzed the serum tolerance experiments with 100% human serum using *A. baumannii* ATCC 17978 and different ST clinical isolates and the results showed that there is no or irregular activity in 100% serum (Supplementary Figure 3).

### Synergistic effect of eAbEndolysin and antibiotics

**Figure 5:**
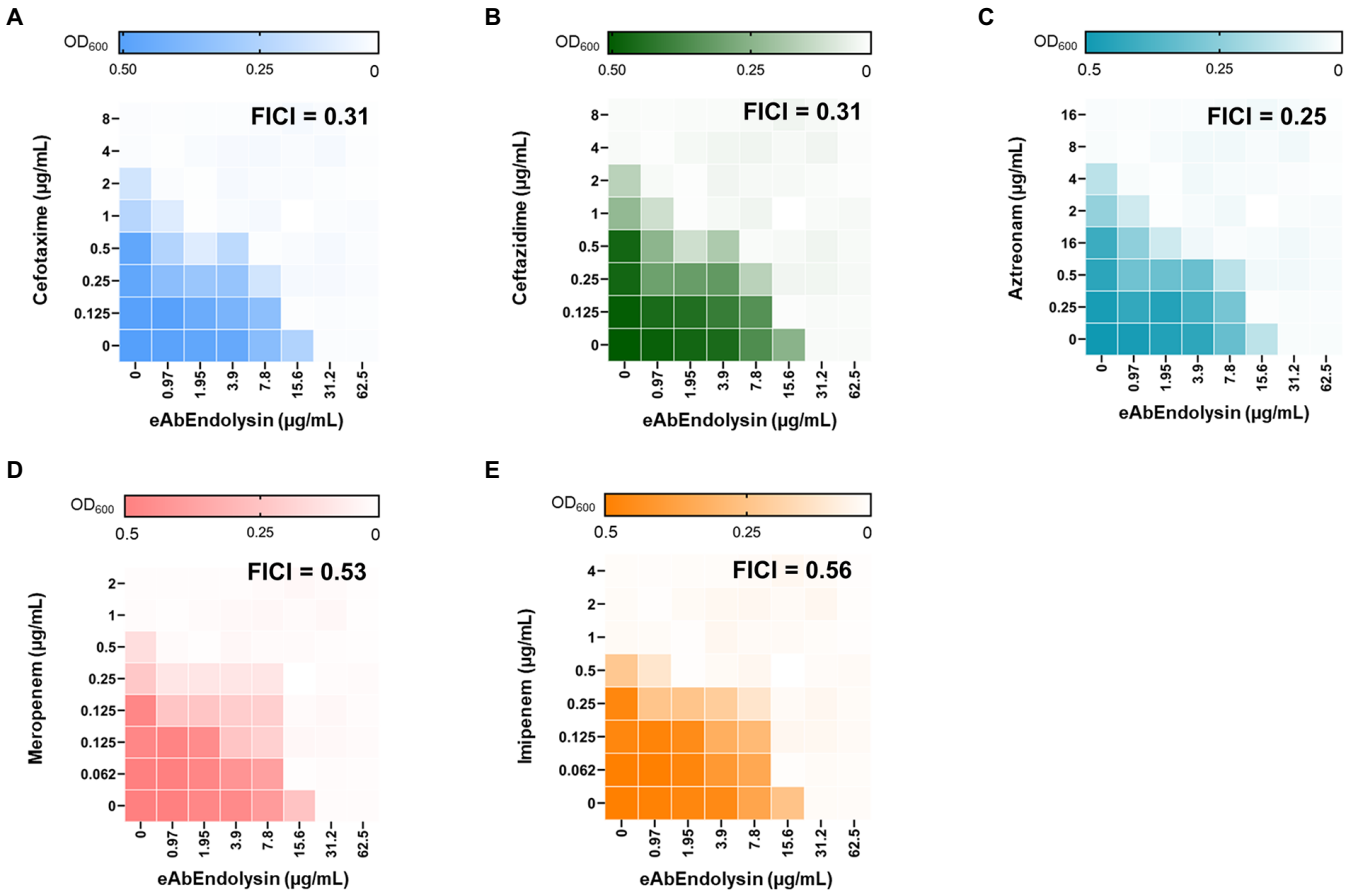
Evaluation of the antimicrobial synergy of eAbEndolysin in *A. baumannii* ATCC 17978 in combination with **(A)** cefotaxime, **(B)** ceftazidime, **(C)** aztreonam, **(D)** meropenem, and **(E)** imipenem. Heat maps of checkerboard titration assays show the optical density of *A. baumannii* at 600 nm. The results are representative of at least three independent experiments.

Engineered endolysins and five different antibiotics that affect bacterial cell wall biosynthesis were screened for their interaction by observing growth inhibition in the checkboard assay. *A. baumannii* 17978 strain was used to determine the combined effects of endolysin, cefotaxime, ceftazidime, aztreonam, meropenem, and imipenem by calculating the FICI. Checkboard assay results showed that the MIC of eAbEndolysin alone was 15.6 μg/ml; however, upon combination with cefotaxime a lower concentration of endolysin exhibited antibacterial activity (Figure 5A). The FICI value for the combination of eAbEndolysin and cefotaxime was determined as 0.31. This indicated a synergistic effect. The FICI values for ceftazidime, aztreonam, meropenem, and imipenem with eAbEndolysin were 0.31, 0.25, 0.53, and 0.56, respectively. Ceftazidime and aztreonam showed synergy with eAbEndolysin (Figures 5B,C) whereas the combination with meropenem or imipenem showed an additive effect (Figures 5D,E). Checkboard assay results using *A. baumannii* clinical strains are listed in Table 1. In case of *A. baumannii* ST191 strain, eAbEndolysin showed synergy effect with cefotaxime, aztreonam, meropenem and imipenem and additive effect with ceftazidime; in ST208 strain synergy was observed with ceftazidime and additive effect with cefotaxime, aztreonam, meropenem and imipenem; in ST229 strain cefotaxime and ceftazidime showed synergy and aztreonam and meropenem showed additive effect; in ST369 strain aztreonam and meropenem showed synergy and cefotaxime, ceftazidime and imipenem showed additive effect; in ST451 strain all 5 tested antibiotics showed synergy effect and in ST784 strain cefotaxime, meropenem and imipenem showed synergy and ceftazidime and aztreonam showed additive effect.

**Table 1:** Fractional inhibitory concentration index (FICI) of eAbEndolysin with various antibiotics.

*Acinetobacter baumannii* clinical isolates	Cefotaxime	Ceftazidime	Aztreonam	Meropenem	Imipenem
KBN10P06070 (ST191)	0.49	0.62	0.49	0.49	0.37
KBN10P04969 (ST208)	0.62	0.37	0.75	0.75	0.53
KBN10P06126 (ST229)	0.37	0.31	0.53	0.56	ND
KBN10P04621 (ST369)	0.62	0.74	0.37	0.37	0.53
KBN10P05231 (ST451)	0.31	0.49	0.49	0.37	0.49
KBN10P05713 (ST784)	0.37	0.56	0.62	0.49	0.49

ND, not determined. Synergistic effect ≤0.5, additive >0.5 to ≤1.

### Cytotoxicity and *in vivo* protection activity of eAbEndolysin

**Figure 6:**
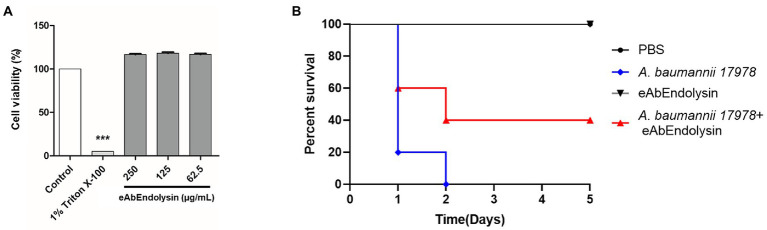
Cytotoxicity and antibacterial efficacy of eAbEndolysin in the mouse systemic infection model. **(A)** Cytotoxicity assay of eAbEndolysin in human alveolar cell line A549. The cells were suspended in 100 μl (5 × 10^3^ cells/well), seeded in 96-well plate, and incubated with eAbEndolysin (final concentration: 250–62.5 μg/ml) for 24 h. PBS and 1% Triton X-100 were used as negative and positive controls, respectively. **(B)** Survival rates for mice infected with *A. baumannii* (2 × 10^8^ CFU). The mice were intraperitoneally injected with PBS (control group), *A. baumannii* ATCC 17978 (infection group), 125 μg/ml of eAbEndolysin (safety test group), or *A. baumannii* +125 μg/ml of eAbEndolysin (treatment group). The mice in each group were treated with eAbEndolysin 0.5 h after infection and monitored for 5 days for post-infection survival. Statistical significance was determined using a one-way ANOVA with multiple comparison across treatment groups. (***p < 0.001).

The cytotoxicity of eAbEndolysin was determined using the WST-1 assay against A549 cells. Cell viability did not decrease after treatment with 62.5, 125, and 250 μg/ml of eAbEndolysin, indicating that eAbEndolysin had no cytotoxic effect (Figure 6A). These results suggest that treatment with eAbEndolysin is safe. We used a systemic infection mouse model to assess the antibacterial efficacy of eAbEndolysin *in vivo*. *A. baumannii* was selected for the protection assay in a murine systemic infection model because eAbEndolysin showed high bactericidal activity against *A. baumannii* strains. Neutropenic mice were IP injected with *A. baumannii*, eAbEndolysin, or PBS and monitored for 5 days post infection. After the bacterial challenge, all the mice succumbed to infection within 2 days of infection. The mice infected with bacteria and treated with 125 μg/ml of eAbEndolysin had 40% survival rate on day 2 and survived until day 5 of infection. The survival rate of the eAbEndolysin-treated group was higher than that of the bacterial infection group, indicating that eAbEndolysin rescued the mice from *A. baumannii* infection. However, mice injected with only PBS survived throughout the experimental period, and similar results were obtained for 125 μg/ml eAbEndolysin-injected mice, indicating the safety of using eAbEndolysin in mice (Figure 6B). A neutropenic mice systemic infection model was used to determine the bacterial loads in different organs after treatment with eAbEndolysin. Total bacterial numbers in the blood and spleen samples were significantly decreased in eAbEndolysin treated group compared to untreated group (Supplementary Figures 1A,D). The bacterial number in lungs and liver samples was also reduced but the result was not significant (Supplementary Figures 1B,C). These results support our *in-vivo* protection assay data for increased survival in eAbEndolysin-treated group mice compared to untreated mice. Another mice survival experiment using endotoxin free eAbEndolysin and AbEndolysin showed that mice infected with bacteria and treated with 125 μg/ml of AbEndolysin or eAbEndolysin had 60% survival rate on day 2 and survived until day 5 of infection. The survival rate of the AbEndolysin and eAbEndolysin-treated group was higher than that of the bacterial infection group, indicating that Endolysin rescued the mice from *A. baumannii* infection. On the other hand, mice infected with bacteria and treated with 250 μg/ml of AbEndolysin or eAbEndolysin had 40% survival rate until day 5 of infection which is lower than 125 μg/ml treated group (Supplementary Figure 4).

## Discussion

The treatment of *A. baumannii* infections with currently available antibiotics is challenging because of their MDR phenotyps. Therefore, an alternative to conventional antibiotics is urgently required. Bacteriophage-derived endolysins offer an option for developing new antimicrobials with novel modes of action. Several lysins are effective against MDR *A. baumannii* (Lai et al., 2011; Lood et al., 2015; Antonova et al., 2019; Wu et al., 2019; Abdelkader et al., 2020; Kim et al., 2020). Gram-negative bacteria possess a protective outer membrane that resists the direct action of endolysin (Fischetti, 2005). We have previously isolated AbEndolysin from phage Ab1656-2, originating from the clinical *A. baumannii* 1,656–2 strain, which has antimicrobial activity against clinical *A. baumannii* strains (Kim et al., 2021). Endolysins engineered by the fusion of different peptides (called Artilysins) can cross the outer membrane barrier of gram-negative bacteria (Briers and Lavigne, 2015). The AMP cecropin A, which has an amphipathic alpha helix and shows antimicrobial activity against gram-negative bacteria, interferes with the outer membrane of the bacteria (Hultmark et al., 1980; Steiner et al., 1988). Fusion of cecropin A with endolysin enhances its permeability to the membrane by distorting the outer membrane, which facilitates cecropin A-fused lysin uptake and subsequently enhances the antibacterial efficacy of the fusion protein (Scott et al., 1999; Hong et al., 2022). Considering these observations, we attempted to elucidate the potential of cecropin A-fused AbEndolysin in this study. Similar approaches have also been used for constructing Artilysins to determine the activity of engineered lysins (Briers et al., 2014; Yang et al., 2015; Defraine et al., 2016; Antonova et al., 2020).

The antibacterial activity (MIC) of eAbEndolysin was 2-8-fold higher than that of the parental AbEndolysin for different *A. baumannii* isolates (Figure 2). In addition to *A. baumannii* ATCC 17978, 12 MDR clinical *A. baumannii* isolates resistant to 10 of the 11 antibiotics tested (Supplementary Table S2) were used. The broth microdilution assay showed that the MIC of AbEndolysin ranged from 62.5 μg/ml to 125 μg/ml, while 15.6–31.2 μg/ml of eAbEndolysin was sufficient for inhibiting the growth of most of the tested strains (Figure 2). N-terminal fusion of cecropin A could have helped AbEndolysin to penetrate the peptidoglycan layer and reach the cell wall for bactericidal action. In this study, we did not evaluate the bactericidal activity of only cecropin A. Previous observations on endolysin engineered by the N-terminal fusion of the truncated peptide cecropin A to endolysins (PlyA and eLysMK34) showed more than 5 log bactericidal activity against *A. baumannii* and *P. aeruginosa*, which supports our present results (Yang et al., 2015; Abdelkader et al., 2020). Our FE-SEM images also showed higher cell lysis capabilities of eAbEndolysin against several clinical *A. baumannii* isolates than those of the parental lysin (Figure 3).

We speculated the influence of temperature, pH, and salinity on eAbEndolysin lysis. The purified protein remained active under 4–42°C. The eAbEndolysin was more active at low pH (5.5–7.5), with the highest activity at pH 7.5. The chimeric endolysin could not tolerate high salinity and showed the highest activity in the absence of NaCl, although it remained active at low NaCl concentrations (Figure 4). Engineered lysin from PlyF307 showed a similar salt-tolerance activity (Thandar et al., 2016). In complex medium such as human serum, significant bactericidal activity of eAbEndolysin was observed until 10% serum compared to native lysin. In high concentration of serum eAbEndolysin loses enzymatic activity which needs further study to reveal which physio-chemical properties needs to improve to increase the serum tolerance.

eAbEndolysin showed synergistic interactions with β-lactam antibiotics against MDR *A. baumannii* isolates. β-lactam antibiotics destroy a β-lactam ring and inhibit penicillin-binding proteins, which help build bacterial cell walls (Cho et al., 2014). *A. baumannii* exhibits resistance to β-lactams by producing extended-spectrum*-*β-lactamases, metallo-β-lactamases, and oxacillinases (Peymani et al., 2011). The combination of cephalosporins (cefotaxime or ceftazidime) and eAbEndolysin may help penetrate the peptidoglycan and allow more lysin to reach the cell membrane. Cefotaxime and ceftazidime showed synergy with eAbEndolysin (Figures 5A,B). Another monobactam antibiotic, aztreonam, also showed synergy with eAbEndolysin (Figure 5C). Two carbapenem antibiotics (meropenem and imipenem) were used to test synergy, and both showed additive effects in combination with eAbEndolysin (Figures 5D,E). Several previous studies have demonstrated the synergistic effects of colistin and endolysin against *A. baumannii* (Thummeepak et al., 2016; Abdelkader et al., 2020, 2022). Although therapy with a combination of multiple antibiotics has been well-documented, very few studies have used a combination of endolysin and β-lactam antibiotics against MDR *A. baumannii*. Thus, this study shows a new therapeutic potential of combination of endolysin with antibiotics for future treatment.

eAbEndolysin (62.5, 125, and 250 μg/ml) did not exhibit cytotoxic effects on A549 cell line and the murine model (Figures 6A,B). Our results showed that 40% *A. baumannii*-infected mice survived after treatment with 125 μg/ml eAbEndolysin, suggesting that eAbEndolysin could treat systemic *A. baumannii* infections (Figure 6B). The number of bacteria in different organs such as blood, lungs, liver and spleen of eAbEndolysin treated mice also reduced compared to untreated mice which supports increased survival rate. In addition, experiment with endotoxin free AbEndolysin and eAbEndolysin (125 μg/ml) treated mice had 60% survival whereas high dose of AbEndolysin or eAbEndolysin (250 μg/ml) treatment showed that mice had 40% survival rate until day 5 of infection which is lower than 125 μg/ml treated group. Rapid lysis of *A. baumannii* by high dose of eAbEndolysin may cause septic shock that might explain the lower survival in high dose treated mice. Although endolysin treatment can reduce bacterial number in different organs, there was no significant difference in survival for treatment groups and dose dependency, the optimum dose of eAbEndolysin in various disease model induced by eAbEndolysin susceptible bacteria should be determined in future studies. Similar studies have shown that lysin LysSS had no cytotoxicity and 40% survival rate against *A. baumannii* infection in murine systemic infection model (Kim et al., 2020). *Acinetobacter baumannii* lysin PlyF307 also showed bactericidal activity and partial protection against *A. baumannii* in bacteremia mouse model (Lood et al., 2015). These reports support the findings of this study.

However, we did not evaluate the bactericidal activity of only cecropin A both *in-vitro* and *in-vivo* conditions. This is one of the limitations of our study to reveal the exact mechanism of action of the chimeric endolysin. In addition, the animal test experiment in this study was preliminary which may vary from different clinical practice results and needs more future study for safety and efficacy of the chimeric protein which needs further experiments.

In conclusion, we constructed eAbEndolysin, an engineered endolysin with improved bactericidal activity. Our results demonstrated that an engineered endolysin has stronger antibacterial activity than that of the parent lysin and showed synergy with β-lactam antibiotics against MDR *A. baumannii* isolates. eAbEndolysin also rescued mice from *A. baumannii* infection in a systemic infection model, suggesting a new solution for treating MDR *A. baumannii* infections.

### Statistical analysis

All experiments in this study were performed independently, and the data are expressed as the mean and standard deviation (SD). All raw data were saved in Excel files and imported into GraphPad Prism software for statistical analysis. Prism (version 9.0) was used to conduct statistical analysis. Student’s t-test and One-way analysis of variance (ANOVA) with multiple comparison across treatment groups (95% confidence interval) was used to determine the significance when needed. A *p*-value <0.05 was considered statistically significant.

## Data availability statement

The original contributions presented in the study are included in the article/Supplementary material, further inquiries can be directed to the corresponding author.

## Ethics statement

The studies involving human participants were reviewed and approved by the human blood experiment that was approved by the Institutional Research Board of Kyungpook National University (KNU-2021-0070). The patients/participants provided their written informed consent to participate in this study. The animal study was reviewed and approved by Prof. Hee Kyung Jin, Department of Laboratory Animal Medicine, College of Veterinary Medicine, Kyungpook National University.

## Author contributions

MI, DK, and MS designed the study. MI and DK wrote the manuscript. MI, DK, S-JP, KK, SA, JeK, SB, C-WH, and MS performed experiments. SK, JuK, and JL reviewed the manuscript. C-WH performed experiment. All the authors have read and agreed to the published version of the manuscript.

## Funding

This research was funded by a grant from the Korea Government National Research Foundation (Grant No. 2022R1A2C2010683) and the Korea Disease Control and Prevention Agency (Grant No. 2022-ER2202-00).

## Conflict of interest

The authors declare that the research was conducted in the absence of any commercial or financial relationships that could be construed as a potential conflict of interest.

## Publisher’s note

All claims expressed in this article are solely those of the authors and do not necessarily represent those of their affiliated organizations, or those of the publisher, the editors and the reviewers. Any product that may be evaluated in this article, or claim that may be made by its manufacturer, is not guaranteed or endorsed by the publisher.
