# Supplementary material for: Engineering of lysin by fusion of antimicrobial peptide (cecropin A) enhances its antibacterial properties against multidrug-resistant Acinetobacter baumannii
Source: Front Microbiol. 2022 Sep 26;13:988522. doi: 10.3389/fmicb.2022.988522 (PMC9549208; doi:10.3389/fmicb.2022.988522)
Supplement: Supplementary file 1 [file Data_Sheet_1.pdf]

Supplementary Figure. 1

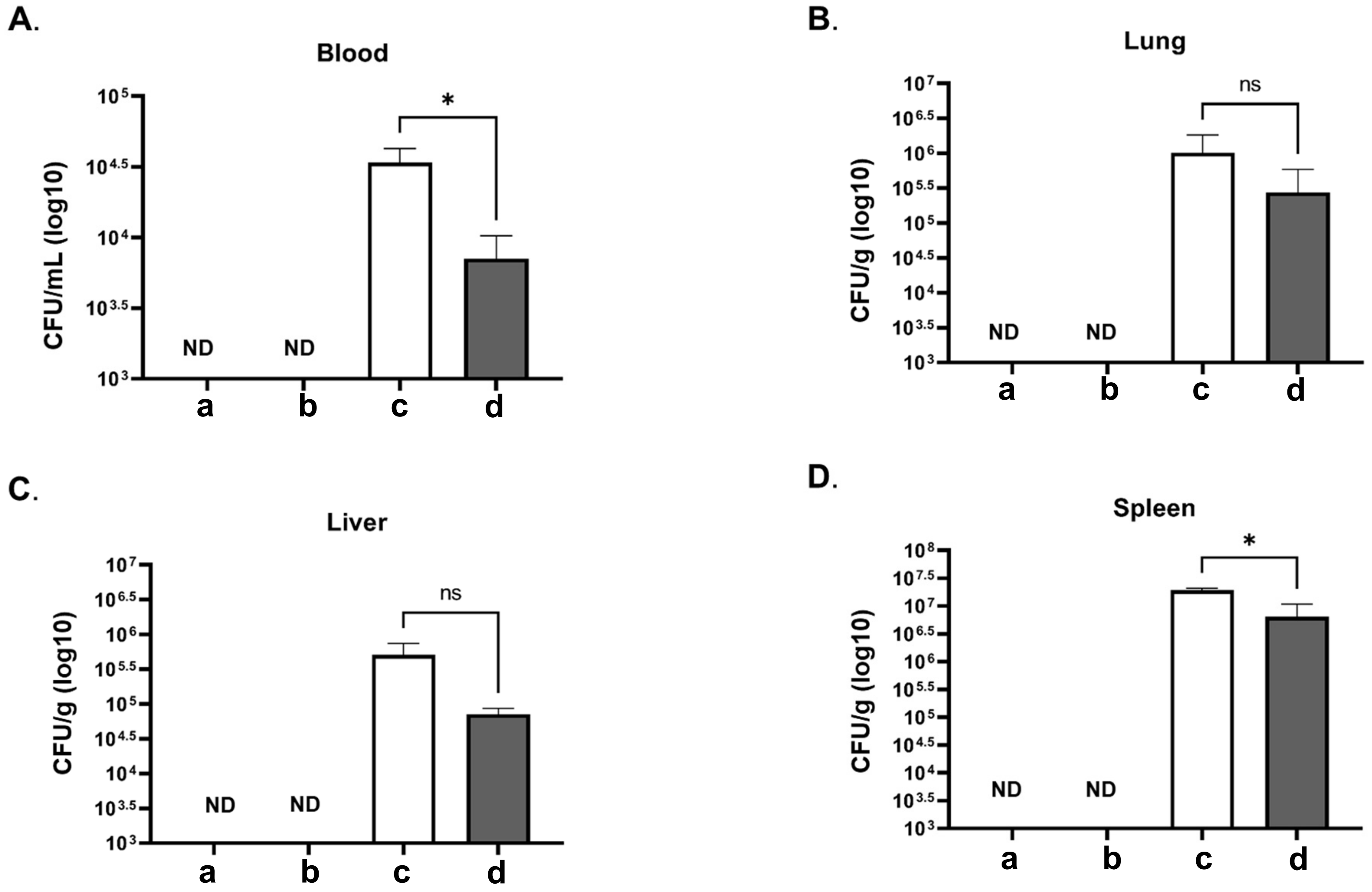

FIGURE S1. Bacterial load of mouse tissues. The mice were divided into four groups (three mice per group) for IP injection as follows: (a) PBS control (b) 125 µg/mL eAbEndolysin safety test (c) infection with *A. baumannii* ATCC 17978 (d) infection with *A. baumannii* + 125 µg/mL eAbEndolysin. Colony forming units (CFU) in mouse (A) Blood (B) Lungs (C) Liver and (D) Spleen tissue. ND= not detected. The data are presented as the mean ± standard deviation. Statistical significance was determined using a one-way ANOVA with multiple comparison across treatment groups. (*** $p < 0.001$)

Supplementary Figure. 2

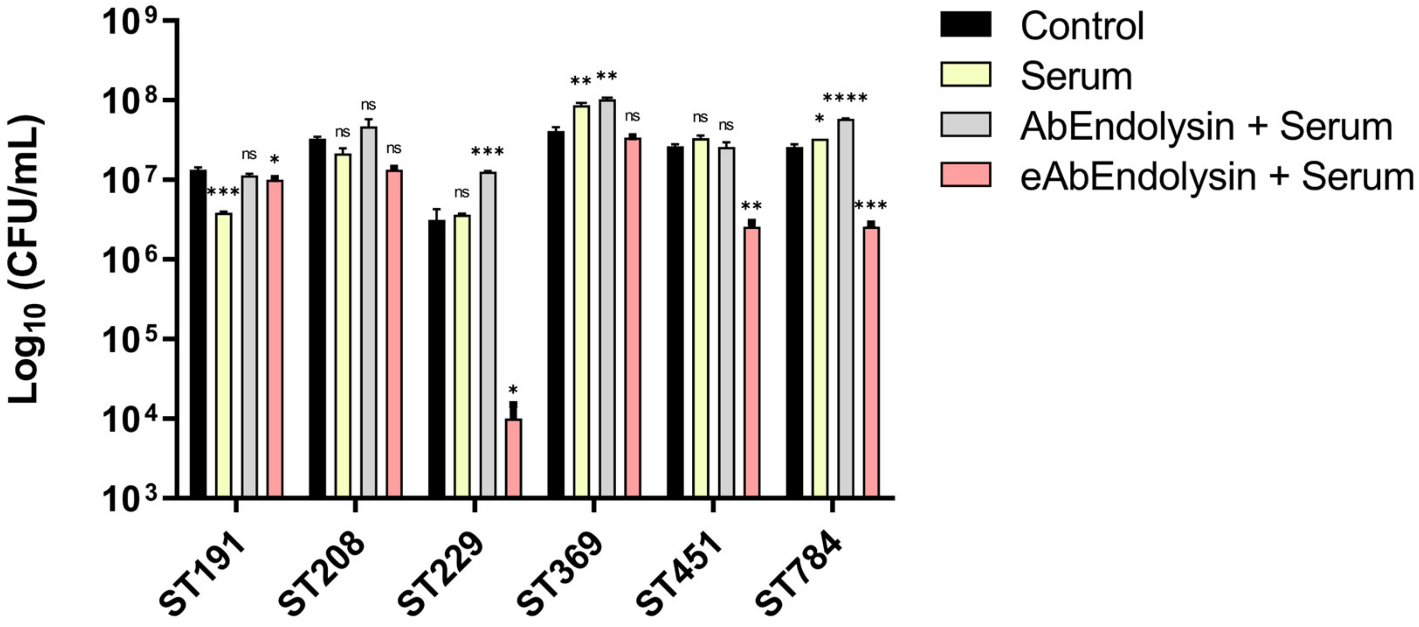

Supplementary Figure. 2: Antibacterial activity of AbEndolysin and eAbEndolysin in presence of human serum. Activity of 250 µg/mL AbEndolysin or eAbEndolysin in presence of 10% human serum using six clinical *A. baumannii* isolates from six different ST-type. The number of log10 CFU/mL was determined through plating 10-fold serial dilution in a bactericidal assay. Data are expressed as the mean ± SD. Experiments were performed independently in duplicate. Statistical significance was determined using a one-way ANOVA with multiple comparison across treatment groups.. (* $p < 0.05$; ** $p < 0.01$; *** $p < 0.001$ and ns- not significant).

Supplementary Figure. 3

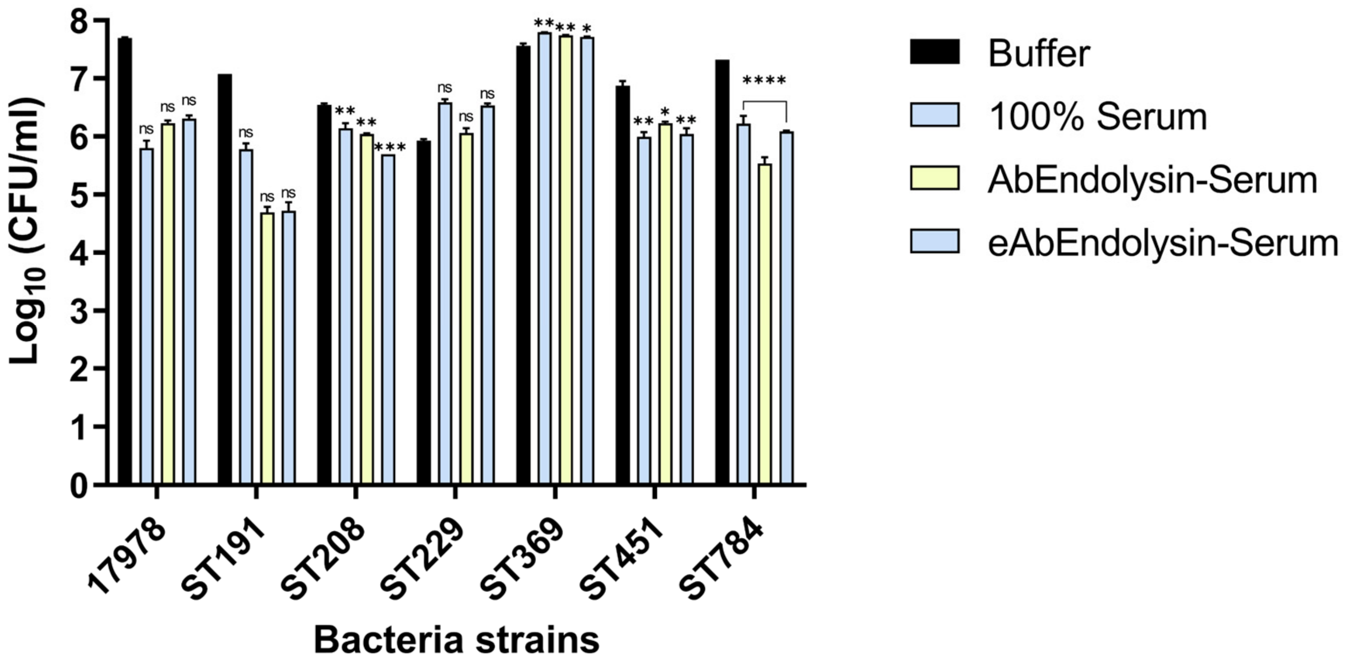

Supplementary Figure. 3: Antibacterial activity of AbEndolysin and eAbEndolysin in presence of 100% human serum. Activity of 125 µg/mL AbEndolysin or eAbEndolysin in presence of 100% human serum using *A. baumannii* ATCC 17978 and six clinical isolates from six different ST-type. The number of log10 CFU/mL was determined through plating 10-fold serial dilution in a bactericidal assay. Data are expressed as the mean ± SD. Experiments were performed independently in duplicate. Statistical significance was determined using a one-way ANOVA with multiple comparison across treatment groups.. (* $p < 0.05$; ** $p < 0.01$; *** $p < 0.001$ and ns- not significant).

Supplementary Figure. 4

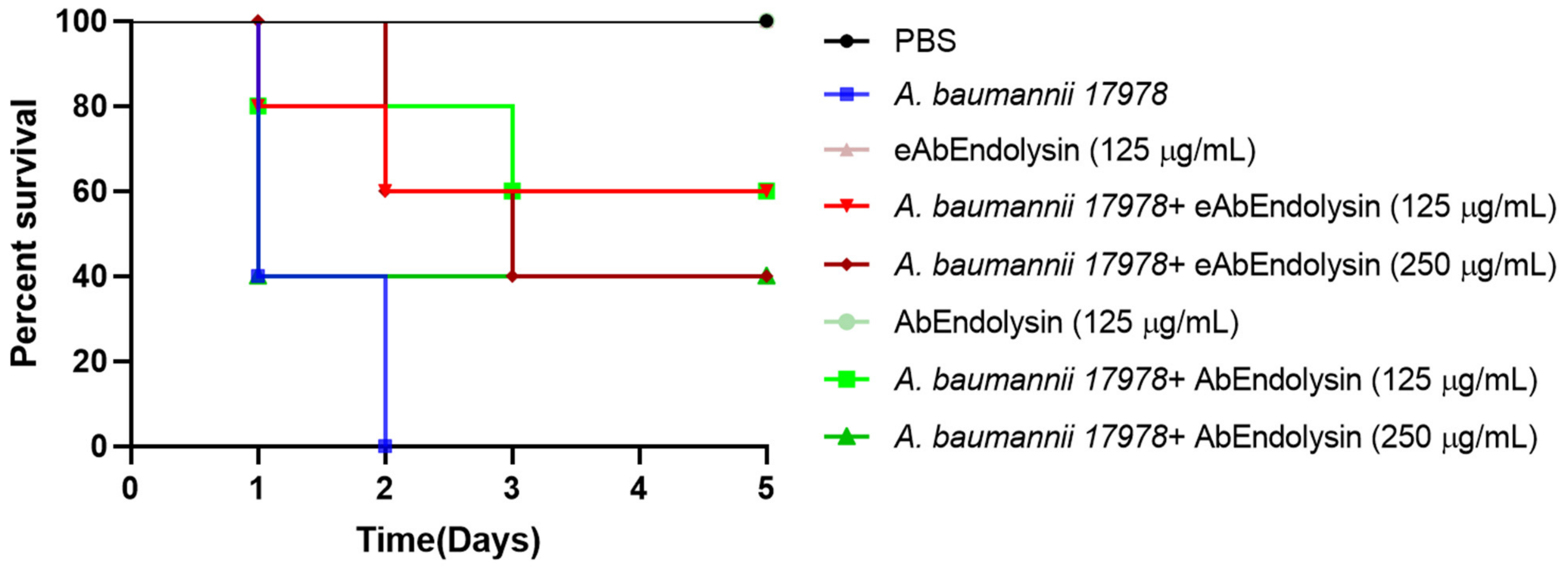

Supplementary Figure. 4: Antibacterial efficacy of eAbEndolysin and AbEndolysin in the mouse systemic infection model.

Survival rates for mice infected with *A. baumannii* (2×10^8 CFU). The mice were intraperitoneally injected with PBS (control group), *A. baumannii* ATCC 17978 (infection group), 125 μg/mL of eAbEndolysin (safety test group), 125 μg/mL and 250 μg/mL eAbEndolysin + infection with *A. baumannii* (treatment group), 125 μg/mL AbEndolysin (safety test group), 125 μg/mL and 250 μg/mL AbEndolysin + infection with *A. baumannii* (treatment group). The mice in each group were treated with eAbEndolysin or AbEndolysin 0.5h after infection and monitored for 5 days for post-infection survival.

Supplementary Figure. 5

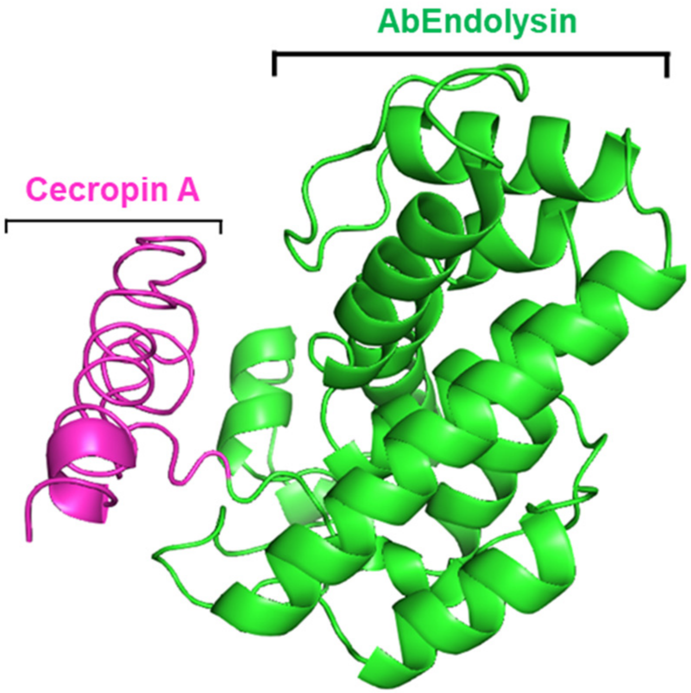

Supplementary Figure. 5: Structure of cecropin A-fused AbEndolysin.

The 3D structure of AbEndolysin-fused cecropin A was predicted using the Phyre program and PyMOL software.

Supplementary document. 1: Nucleotide sequence of cecropin A fused AbEndolysin.

CecropinA fused AbEndolysin

ATGGCTAGCAAATGGAAACTGTTTAAAAAAATTGAAAAAGTGGGCCAGAACATTCGCGATGGCA
TTATTAAAGCGGGCCCGGCGGTGGCGGTGGTGGGCCAGGCGACCCAGATTGCGAAAGGCAGCGG
CTCGGGTAGTATGCCGCCTTCGGGCGGTTTTTTACATCTGAAGGAAACCGAAATGAACATTGAA
CAATATCTGGACGAGTTAATTAAGCGTGAGGGCGGGTACGTAAACAACCCAGCAGATCGAGGCG
GTGAAACAAAGTACGGTATTACTGAAGCAGTAGCACGTACTAACGGCTTTAAGGGCAACATGAA
AGATTTACCGCTTGATGTGGCCAAAGCCATTTATAAAAAGCAGTATTGGACAGATCCGCGATTT
GATCAAGTGAATGTAATTAGCTCGTTAGTTGCTGAAGAGCTTTTAGATACTGGGGTAAATTGCG
GTACCGGATTTGCAAAACCACTCTTACAGCGTGCTTTAAATTTGCTGAATAACCAAGGTAAAGC
AGGTTGGCCAGATTTAACAGTTGACGGAATTTATGGTCCAGCAACTCTTAATGCACTCAAAACT
TATCTGGCCAAGCGTGGAAAAGACGGCGAAAAAGTCCTGGTGCGTGTTCTTAATATCATGCAAG
GGCAACGTTACATTGAAATCTGTGAACGCAATCCTAGCCAGGAACAGTTTTTCTATGGTTGGAT
CGCCAATCGAGTTGTTATATGA

Red color indicates nucleotide sequence of Cecropin A; Yellow color indicates start and end of AbEndolysin sequence as well primer sequence.

Table S1. Bacterial strains and plasmids used in this study.

Bacteria or plasmids	Relevant characteristics	Reference or source
**Bacterial strains**		
A. baumannii		
ATCC 17978	Wild-type strain	Lab stock
KBN10P04948	Clinical strain	Laboratory collection
KBN10P06070	Clinical strain	Laboratory collection
KBN10P04320	Clinical strain	Laboratory collection
KBN10P04969	Clinical strain	Laboratory collection
KBN10P04598	Clinical strain	Laboratory collection
KBN10P06126	Clinical strain	Laboratory collection
KBN10P04596	Clinical strain	Laboratory collection
KBN10P04621	Clinical strain	Laboratory collection
KBN10P05231	Clinical strain	Laboratory collection
KBN10P05986	Clinical strain	Laboratory collection
KBN10P04697	Clinical strain	Laboratory collection
KBN10P05713	Clinical strain	Laboratory collection
E. coli		
DH5α *λ pir*	*supE44 DlacU*169 *(f80 lacZ DM15) hsdR17 recA1 endA1 gyrA 96 thi-1 relA1 λ* pir phage lysogen; plasmid replication	Laboratory collection
BL21 star (DE3)	*F-ompT hsdSB* ($rB^{-}mB^{-}$) *gal dcm rne13*(DE3)	Laboratory collection
BL21 star (DE3):: pB4: Cecropin A-AbEndolysin	pB4 with Cecropin A-AbEndolysin transformed in *E. coli*	This study

E. coli ATCC 25922	Quality control strain for MIC	Laboratory collection
P. aeruginosa ATCC 27853	Quality control strain for MIC	Laboratory collection
**Plasmids**		
pB4	derived from pET21a, HisTag-MBP, TEV cleavable	Laboratory collection
pB4::Cecropin A-AbEndolysin	pB4 with Cecropin A-AbEndolysin	This study

Table S2: Clinical *A. baumannii* strains antibiotic susceptibility

Bacterial Strains	Specimens	MLST	Antibiotics										
			Ampicillin/ Sulbactam	Aztreonam	Cefotaxime	Ceftazidime	Cefepime	Imipenem	Meropenem	Piperacillin	Piperacillin/ Tazobactam	Ticarcillin/ Clavulanic	Colistin
KBN10P04948	Blood	191	R	R	R	R	R	R	R	R	R	R	S
KBN10P06070	Sputum	191	I	R	R	R	R	R	R	R	R	R	S
KBN10P04320	CSF	208	R	R	R	R	R	R	R	R	R	R	S
KBN10P04969	GI	208	R	R	R	R	R	R	R	R	R	R	S
KBN10P04598	Blood	229	R	R	R	R	R	R	R	R	R	R	S
KBN10P06126	Blood	229	R	R	R	R	R	R	R	R	R	R	S
KBN10P04596	Sputum	369	R	R	R	R	R	R	R	R	R	R	S
KBN10P04621	Blood	369	R	R	R	R	R	R	R	R	R	R	S
KBN10P05231	Blood	451	R	R	R	R	R	R	R	R	R	R	S
KBN10P05986	Blood	451	R	R	R	R	R	R	R	R	R	R	S
KBN10P04697	Blood	784	S	R	R	R	R	R	R	R	R	R	S
KBN10P05713	Sputum	784	I	R	R	R	R	R	R	R	R	R	S

CSF= Cerebrospinal fluid, GI= Gastrointestinal tract, R= Resistant, I= Intermediate, and S= Susceptible
